# The Effect of Fatigue on Single-Leg Postural Sway and Its Transient Characteristics in Healthy Young Adults

**DOI:** 10.3389/fphys.2021.720905

**Published:** 2021-08-18

**Authors:** Žiga Kozinc, Nebojša Trajković, Darjan Smajla, Nejc Šarabon

**Affiliations:** ^1^Faculty of Health Sciences, University of Primorska, Izola, Slovenia; ^2^Andrej Marušič Institute, University of Primorska, Koper, Slovenia; ^3^Faculty of Sport and Physical Education, University of Niš, Niš, Serbia; ^4^Human Health Department, InnoRenew CoE, Izola, Slovenia; ^5^Laboratory for Motor Control and Motor Behavior, S2P, Science to Practice, Ltd., Ljubljana, Slovenia

**Keywords:** body sway, posture, stability, transient behavior, fatiguing

## Abstract

Neuromuscular fatigue is known to impair balance ability, which is reflected in increased postural sway during quiet standing tasks. Recently, quantifying transient characteristics of postural sway has been suggested as an approach to obtain additional information regarding postural control. However, this approach is currently vastly unexplored. The purpose of this study was to investigate the effects of fatigue (induced by a repeated change of direction task) on postural sway and its transient characteristics during single-leg standing, including whole-trial estimates and indexes of transient behavior in young healthy active adults. The study involved 28 physically active students (14 females). Single-leg postural sway was recorded for 30s before and after a fatiguing protocol, which consisted of a repeated change of direction tasks. We calculated the traditional whole-trial estimates of postural sway [center-of-pressure (CoP) velocity and amplitude in anterior-posterior (AP) and medial-lateral (ML) directions] and corresponding transient behavior indexes, based on three 10-s intervals. Statistically significant sex×fatigue interaction with medium effect sizes was found for whole-trial CoP velocity in AP (*p*=0.028; *η*^2^=0.17) and ML directions (*p*=0.019; *η*^2^=0.19). *Post-hoc* test showed that both variables substantially decreased in female participants (*p*=0.041–0.045; *d*=0.54–0.56), but remained similar in males (*p*=0.194–0.294). There were small to medium statistically significant main effects of fatigue on transient index for CoP amplitude in both directions (*p*=0.042–0.049; *η*^2^=0.02–0.14). Notably, CoP AP amplitude increased in the first 10-s interval for males (before fatigue: 5.6±1.3mm; after fatigue: 6.3±1.6mm), while the CoP AP amplitude in the third interval remained similar after fatigue (before fatigue: 5.5±1.4mm; after fatigue: 5.1±1.2mm). In conclusion, the responses to fatigue in terms of postural sway were time interval specific, and there were certain sex-differences in responses to fatigue, which could be related to better ability to adapt balance strategies in females. Moreover, our results demonstrate that the indexes of transient behavior could perhaps detect smaller fatigue-induced changes in postural sway that are seen in whole-trial estimates.

## Introduction

Balance and postural control are fundamental abilities underpinning normal human movement function (Pollock et al., 2000; Ivanenko and Gurfinkel, 2018). Assessments of balance are routinely performed in research and clinical practice, studying patients with neuromuscular impairments (Gunn et al., 2015; Shen et al., 2016), children (Geldhof et al., 2006; Alsakhawi and Elshafey, 2019), and older adults (Park, 2018; Kozinc et al., 2020). In athletes, dynamic balance tests have been used to assess injury risk (De Noronha et al., 2006; Plisky et al., 2006); however, the utility of static balance tests (e.g., assessment of postural sway during quiet standing) for athletic populations has not been explored as much. In a literature review, Hrysomallis (2011) reported that static and dynamic balance are related to athletic performance and that balance exercise can contribute to improvements in the performance of movement tasks such as jumping and agility tests. Clearly, the role of balance assessment should not be neglected in athletes, both from injury prevention and performance enhancement aspects. It has been postulated that neuromuscular fatigue, owing to its effects on movement biomechanics, plays an important role in injury risk in sport (Barber-Westin and Noyes, 2017; Benjaminse et al., 2019; Bourne et al., 2019). Therefore, it could be of particular interest to understand how neuromuscular fatigue affects balance.

Static balance is traditionally assessed by measuring postural sway during quiet stance, most typically through center-of-pressure (CoP) movement recordings. The negative effect of neuromuscular fatigue on balance is very well-known. These effects may be driven by various mechanisms, such as altered position sense (Forestier et al., 2002), changes in visual, vestibular, and proprioceptive sensory inputs (Lepers et al., 1997), and reductions in muscle output (Enoka and Stuart, 1992); for review, see Paillard (2012). Increases of postural sway during parallel and single-leg stance have been documented after locally fatiguing lower limb (Lin et al., 2009; Bisson et al., 2011; Zech et al., 2012; Boyas et al., 2013; Barbieri et al., 2019) and after fatiguing the trunk muscles (Pline et al., 2006; Lin et al., 2009; Ghamkhar and Kahlaee, 2019). Similarly, postural sway during single-leg stance is increased after global fatiguing protocols, based on running (Zech et al., 2012; Steib et al., 2013; Beurskens et al., 2016), rowing (Springer and Pincivero, 2009), load lifting (Bannon et al., 2018), and repeated sprinting (Pau et al., 2014). In addition, isolated fatiguing of inspiratory muscles was also reported to result in increased postural sway during parallel stance (Janssens et al., 2010). Since sports training and competitions mostly include full-body movement tasks, it seems reasonable to assume that investigating the effect of global fatigue on balance has greater ecological validity and practical relevance for athletes. In the study performed by Zech et al. (2012), the global fatigue had a significantly larger effect on single-leg postural sway (+47%) than local fatigue (+10%), whereas similar impairments in dynamic balance (assessed through star excursion balance test) were observed after local and global fatigue in another study (Garcia-Gallart and Encarnacion-Martinez, 2019). Greig and Walker-Johnson (2007) investigated the effect of a 90-min running protocol on single-leg postural sway in male soccer players and reported that postural sway was not meaningfully affected. On the other hand, an increase in single-leg postural sway was produced by a zig-zag task in male and female athletes from field-based team sports (Lacey and Donne, 2019). Interestingly, aerobic fatigue had a more pronounced effect on single-leg postural sway than anaerobic fatigue in a study conducted on female soccer players (Güler et al., 2020). Recently, Bedo et al. (2020) used a complex fatiguing protocol, consisting of multiple tasks, such as jumping, sidestep cutting, sprinting, and landing. This protocol resulted in a large increase in single-leg postural sway, along with a decrement in maximal strength. Both abilities, however, returned to baseline within 5min (Bedo et al., 2020). On the contrary, Baghbani et al. (2016) observed no effect of a complex seven-station fatigue protocol on dynamic balance (star excursion test in athletes), while the non-athlete control group did perform worse after fatiguing as expected.

The evidence regarding the differences between the sexes in terms of postural sway are somewhat ambiguous, as previous experiments showed either no differences between males and females (Cruz-Gómez et al., 2011; Olchowik et al., 2015) or that females exhibit lower postural sway (Greve et al., 2013; Sell et al., 2018). Caution is needed when examining sex differences, as anthropometric variables may cofound the results (Era et al., 1996; Bryant et al., 2005; Villarrasa-Sapiña et al., 2016; Plandowska et al., 2019). Studies have also suggested that postural sway in males is more sensitive to fatigue (Bannon et al., 2018) and vision elimination (Masui et al., 2005). Therefore, when examining the effects of fatigue on postural sway, sex, and anthropometric variables should be considered as factors in statistical analyses.

Having in mind that balance has been linked to sports performance (Hrysomallis, 2011) and injury risk (Hrysomallis, 2007); the effect of fatigue-induced balance deterioration on injury risk and sports performance is unknown. Recently, a new approach to analyze transient behavior of CoP data has been proposed by Reed et al. (2020). Instead of averaging the CoP data across the trial, this approach separates the data into several time intervals and evaluates the changes in CoP behavior among these intervals. Reed et al. (2020) reported that indexes of the transient behavior of postural sway were independent of the whole-trial estimates (in other words, they were not correlated with the whole-trial estimates), and were sensitive to age and vision elimination. In our subsequent studies, we confirmed the independence of transient indexes and the whole-trial variables, and demonstrated their sensitivity (albeit with small effect sizes) to leg preference (Kozinc and Šarabon, 2021a). Moreover, we found different transient behavior (i.e., quicker reduction of postural sway throughout the trial) in ballet dancers compared to young adults (Kozinc and Šarabon, 2021b). Admittedly, averaging the data is superior in terms of the reliability of the outcomes, however, indexes of transient behavior provide additional information regarding an individual’s balance control that are masked (e.g., initial less stable period) by averaging the whole-trial data. This approach could be relevant for injury risk screening in sport. However, we found no differences in terms of the transient behavior of postural sway in single-leg stance between athletes with and without ankle sprain history (Kozinc et al., 2021). In this study, we investigated whether indexes of transient behavior of single-leg postural sway could be used to detect deterioration in balance, induced by fatigue. Since fatigue is a major determinant of injury risk in athletes (Benjaminse et al., 2019; Bourne et al., 2019), sensitive and valid approaches to monitor it, are needed. Indexes of transient behavior could be particularly useful if they showed higher sensitivity to fatigue in comparison to whole-trial estimates postural sway. Therefore, the purpose of this study was to investigate the effects of whole-body fatigue on postural sway during single-leg standing, including whole-trial estimates and indexes of transient behavior in young healthy active adults. Based on the large body of literature on the effects of fatigue on postural sway, we considered the single-leg stance as the most appropriate task, being more challenging than bipedal tasks and consequently being expected to be more sensitive to fatigue. We hypothesized that the fatiguing protocol will result in increased whole-trial CoP amplitude and velocity. The lack of previous studies precluded a formation of hypothesis regarding the effects of fatigue on indexes of transient behavior. The secondary aim of this study was to examine if the effects of fatigue on postural sway are sex-specific.

## Materials and Methods

### Participants

Based on the previous studies (Zech et al., 2012; Steib et al., 2013; Pau et al., 2014; Lacey and Donne, 2019; Güler et al., 2020), we expected to observe an increase in CoP velocity and amplitude with an effect size (Cohen’s *d*) of ~0.75–1.00. We calculated that 19 participants would be needed to confirm such an effect with 90% statistical power and alpha level of 0.05. However, since the fatiguing protocols as well as postural tasks were different in previous studies, we aimed for a higher sample size to detect potential smaller effect. Thus, the study sample comprised of 28 kinesiology students (14 females, age: 22.1±2.1years; body mass: 60.5±5.7kg; body height: 166.9±8.4cm; body mass index: 21.8±2.4; and 14 males, age: 26.0±5.1years; body mass: 76.6±6.7; body height: 181.5±4.6cm; body mass index: 24.2±2.1). The participants were required to be free from musculoskeletal injuries in the previous 6months, pain and other medical conditions that could affect postural control. All participants reported being physically active at least three times a week, with different preferred exercises of choice (e.g., resistance training and aerobic exercise). They also completed several study courses that required them to exhibit decent levels of aerobic endurance, strength, flexibility, and balance. For instance, they were required to reach the norms for 2,400m run test (males: <10min 30s; females: <11min 30s), hamstring flexibility [reaching to the floor with fingertips (males) or full palms (females) from a standing position, without flexing the knees], and full body strength (lifting 50% of their body weight three times with an overhead squat exercise technique), and correctly perform several drills or movement skills (swimming techniques, basic elements of soccer, volleyball, basketball and handball, and athletic drills). The procedures of this study were approved by National Medical Ethics Committee (approval number: 0120-690/2017/8) and were conducted in accordance to the Declaration of Helsinki. After the explanation of the experimental procedures, a written informed consent was obtained from all participants.

### Study Design and Experimental Tasks

The study was conducted in a single session, lasting approximately 1h. Before the measurements, the participants performed a 15-min warm up, consisting of 10min of light-intensity running on an indoor track and 5min of dynamic stretching exercises. Postural sway was assessed during single-leg quiet stance before and after the fatiguing protocol (see below), using a force plate positioned on a parquet floor. Before the main trials, a standardized 20s familiarization trial was performed to introduce the task requirements to the participants. For the main trials, the participants were required to maintain a single-leg stance for 30s. The knee of the stance leg was in an extended position, but it was not allowed to completely lock (i.e., hyperextend). The hip of the other leg was in a neutral position (0°), whereas the knee was flexed at 90° and was not allowed to be touching the standing leg. Participants looked at a fixed point at an eye level and ~3m away from them. The hands were placed on the hips throughout the trials. A 30-s repetition was performed before and after the fatigue. The participants assumed the single-leg position, and the examiner started the acquisition after ~1s. After the fatiguing protocol was finished, the postural sway assessments were repeated immediately. We analyzed the postural sway on the preferred leg, which was determined as the leg that the participant would use to kick a ball. It has been shown that postural sway is slightly smaller during standing on a preferred leg compared to the non-preferred leg (Kozinc and Šarabon, 2021a).

### Fatiguing Protocol

The fatiguing protocol consisted of two repeated change-of-direction (CoD) tasks. Both tasks encompassed a 5+5m run with either a 90 or 180° CoD maneuvers in between, and were performed in both left and right directions. These procedures are producing reliable outcomes (ICC=0.84–0.96; inter-trial variation <2%) in our laboratory (Šarabon et al., 2020). The protocol was performed in a gym (parquet floor). The time to complete each repetition was measured with photocell timing gates (Brower Timing Systems, Draper, UT, United States). The gates were placed at about hip height, with the start line being 0.5m behind the first timing gate to avoid early triggering. Before the fatiguing protocol, the participants first completed two familiarization trials for each task (two trials for left and right directions each) at 50 and 75% of their maximal effort. Then, they performed three repetitions with the maximal effort for each task to establish baseline times. The fatiguing protocol involved performing the CoD tasks in an alternating order (e.g., 90° – left, 90° – right, 180° – left, and 180° – right), with 5s breaks in between. The protocol was stopped when the time to complete the task increased by 20% or more in the 180° turn task for the dominant direction (the direction for which shorter time was recorded at baseline). Right after the protocol stopped, the participants were asked to report their rate of perceived exertion using a 0–10 scale as suggested by Foster et al. (2001).

### Data Processing and Outcome Measures

The ground reaction force data were collected (sampling frequency: 1,000Hz) by a force platform (model 9260AA; Kistler, Winterthur, Switzerland) and were low-pass filtered (Butterworth, second order, 10Hz), using the manufacturer’s MARS Software (Kistler, Winterthur, Switzerland). The data were then processed in the same software in order to obtain CoP velocity [total, anterior-posterior (AP) and medial-lateral (ML)], CoP amplitude (AP and ML), and CoP frequency. The CoP amplitude was defined as the mean amount of the CoP sway in AP or ML direction, calculated as the common length the COP sway trajectory only in the given direction, divided by the number of changes of movement direction. Firstly, we calculated the traditional whole-trial estimates (i.e., averaged CoP characteristics over the whole 30s of the trial). In addition, we also calculated the relative differences between the first and the second (DIF_21) and first and third (DIF_31) 10-s time intervals within the whole trial. For each interval, that data were locally demeaned. The relative differences between the intervals were expressed as percentages (100% representing no change; >100% indicating an increase in time; and <100% indicating a decrease in time).

### Statistical Analysis

Statistical analyses were done in SPSS (version 25.0; SPSS Inc., Chicago, United States). Descriptive statistics were calculated and reported as mean±SD. The normality of the data distribution was verified with Shapiro-Wilk tests (all *p*≤0.065). A two-way ANOVA with one between-participant factor (i.e., sex) and one within-participant factor (fatigue) was run to explore the effects of sex, fatigue, as well as sex×fatigue interaction for CoP variables and indexes of transient behavior (i.e., the DIF_21 and DIF_21). The effect sizes were expressed as partial eta-squared (*η*^2^) and interpreted as small (<0.13), medium (0.13–0.26), and large (>0.26; Bakeman, 2005). Bonferroni-corrected *t*-test were used to explore the effect of sex separately before and after fatigue, the effects of fatigue for each sex, and the effects of sex on rating of perceived exertion and differences fatiguing protocol duration between the sexes. The effect sizes for *t*-tests were calculated as Cohen’s *d* (0.0–0.2 – trivial; 0.2–0.6 – moderate; 0.6–1.2 – large; >1.2 – very large; Bernards et al., 2017). We also examined the associations between whole-trial variables and corresponding indexes of transient behavior (i.e., the DIF_21 and DIF_21) before and after the fatigue. Correlations were assessed by Pearson’s correlation coefficients and interpreted as negligible (<0.1), weak (0.1–0.4), moderate (0.4–0.7), strong (0.7–0.9), and very strong (>0.9; Schober and Schwarte, 2018). For all analyses, the threshold for statistical significance was set at *p*<0.05.

## Results

There were statistically significant differences between the sexes regarding body height (*t*=6.1; *p*<0.001), body mass (*t*=8.6; *p*<0.001), and body mass index (*t*=2.9; *p*=0.007).

### Pre-fatigue Analyses

In the pre-fatigue state, there was a statistically significant moderate correlation between whole-trial CoP ML velocity and its DIF_21 index (*r*=0.52; *p*=0.005). This association was also statistically significant when assessed separately for females (*r*=0.61; *p*=0.018), but not for males (*r*=0.43; *p*=0.125). The associations between the remaining variables and their corresponding DIF_21 or DIF_31 were not statistically significant, regardless if whole sample or separate gender subgroups were analyzed (*r*=0.09–0.33; *p*=0.090–0.667). Before the fatigue, the difference between the genders was only observed for whole-trial CoP amplitude in AP (*p*=0.041; *d*=0.71) and ML direction (*p*=0.023; *d*=0.91), with the females exhibiting lower values (see Table 1 for descriptive statistics). However, when CoP variables were normalized to body height or body mass, there were no differences between the sexes (*p*=0.158–0.434 and 0.112–0.496, respectively). There were no statistically significant correlations between height and CoP variables (*r*=0.03–0.31; *p*=0.095–0.893). None of the transient behavior indexes was different between sexes (all *p*≤0.288).

**Table 1:** Postural sway variables and their transient behavior indexes before and after the fatiguing protocol.

Outcome variable	Before fatigue				After fatigue				Effect size (*η*^2^)		
	Male		Female		Male		Female				
	Mean	SD	Mean	SD	Mean	SD	Mean	SD	Gender	Fatigue	Interaction
CoP VEL AP (mm/s)	27.8	3.9	28.3	6.4	29.7	5.0	24.8	5.8	0.06	0.02	0.17^*^
CoP VEL ML (mm/s)	32.2	5.4	31.2	6.3	34.5	7.3	27.8	6.0	0.12	0.01	0.19^*^
CoP AMP AP (mm)	5.6	1.5	4.7	0.9	5.6	1.0	4.1	1.0	0.34^*^	0.06	0.06
CoP AMP ML (mm)	7.9	2.1	6.3	1.2	8.1	2.6	5.4	1.6	0.30^*^	0.03	0.07
CoP VEL AP – DIF_21 (%)	100.1	22.3	105.3	24.8	94.1	20.3	92.1	16.1	0.00	0.12	0.02
CoP VEL AP – DIF_31 (%)	93.4	14.4	92.1	16.1	86.4	17.9	88.0	21.6	0.00	0.05	0.00
CoP VEL ML – DIF_21 (%)	97.9	18.7	102.8	20.4	98.8	24.7	87.4	18.5	0.01	0.10	0.12
CoP VEL ML – DIF_31 (%)	88.6	18.0	93.0	24.4	90.1	28.1	85.6	15.5	0.00	0.01	0.02
CoP AMP AP – DIF_21 (%)	107.6	38.9	109.6	27.0	96.3	33.7	91.2	18.6	0.00	0.11	0.01
CoP AMP AP – DIF_31 (%)	99.6	18.5	92.3	17.0	83.8	27.9	83.2	22.9	0.02	0.14^*^	0.01
CoP AMP ML – DIF_21 (%)	101.7	26.3	107.2	24.7	94.5	30.5	85.9	26.1	0.00	0.14^*^	0.04
CoP AMP ML – DIF_31 (%)	95.2	29.4	93.7	36.5	90.6	39.5	84.9	27.8	0.01	0.02	0.00

CoP, centre of pressure; VEL, velocity; AMP, amplitude; AP, anterior-posterior; ML, medial-lateral; DIF_21, relative differences between the first and the second 10-s time intervals within the whole trial; and DIF_31, relative differences between the first and the third 10-s time intervals within the whole trial.

* Denotes statistically significant effect (p < 0.05).

### Effects of Fatigue on Whole-Trial Estimates

After the fatiguing protocol, the average rating of perceived exertion score was 8.7±0.8 and was not statistically significantly different (*p*=0.376) between males (9±1) and females (9±1). On average, the males and the females performed 30.8±6.1 and 28.8±6.5 CoD maneuvers, respectively, with the group difference not being statistically significant (*p*=0.411). The descriptive statistics for all outcome variables related to postural sway before and after fatigue is available in Table 1. Statistically significant gender×fatigue interaction with medium effect sizes was found for whole-trial CoP velocity in AP (*p*=0.028; *η*^2^=0.17) and ML directions (*p*=0.019; *η*^2^=0.19). Both interactions were still present when the outcomes were normalized to body height (*p*=0.018–0.027; *η*^2^=0.17–0.19) or body mass (*p*=0.015–0.019; *η*^2^=0.19–0.21). *Post-hoc* test showed that the both variables substantially decreased in female participants (*p*=0.041–0.045; *d*=0.54–0.56), but remained similar in males (*p*=0.194–0.294). Although not reaching statistical significance, there were also notable decreases in CoP AP and ML amplitude in females (*p*=0.056–0.058; *d*=0.61–0.62).

### Effects of Fatigue on Transient Behavior of Postural Sway

None of the transient behavior indexes showed statistically significant gender×fatigue interaction. There were small to medium statistically significant main effects of fatigue for DIF_31 for CoP AP as well as DIF_21 for CoP ML amplitude (*p*=0.042–0.049; *η*^2^=0.02–0.14). The fatigue decreased the values of DIF_31 and DIF_21, which suggests that after fatigue, the CoP amplitude dropped to a larger extent throughout the 30-s trial. *Post-hoc t*-tests revealed that DIF_31 for CoP AP amplitude was decreased after fatigue in males (*p*=0.044; *d*=0.65), but not in females (*p*=0.310). Looking at the descriptive statistics for interval-specific data for males (provided in Table 2) reveals that the effect of fatigue on in DIF_31 was as a result of an increased CoP AP amplitude in the first 10-s interval in males (before fatigue: 5.6±1.3mm; after fatigue: 6.3±1.6mm), while the CoP AP amplitude in the third interval was slightly decreased after fatigue (before fatigue: 5.5±1.4mm; after fatigue: 5.1±1.2mm).

**Table 2:** Interval-specific descriptive statistics.

Outcome variable	Before fatigue				After fatigue			
	Male		Female		Male		Female	
	Mean	*SD*	Mean	*SD*	Mean	*SD*	Mean	*SD*
CoP VEL AP first interval (mm/s)	28.7	4.5	28.7	6.2	32.5	8.8	26.6	5.5
CoP VEL AP second interval (mm/s)	28.3	5.4	30.1	9.1	29.6	5.7	24.5	6.7
CoP VEL AP third interval (mm/s)	26.5	4.2	26.2	6.2	27.0	3.6	23.3	7.1
CoP VEL ML first interval (mm/s)	34.0	5.8	31.9	5.9	36.2	6.8	30.6	5.8
CoP VEL ML second interval (mm/s)	33.0	7.3	32.7	8.8	35.5	9.8	26.8	7.7
CoP VEL ML third interval (mm/s)	29.7	5.9	29.0	7.7	31.9	8.6	26.0	6.2
CoP AMP AP first interval (mm)	5.6	1.3	4.8	1.0	6.3	1.6	4.5	1.0
CoP AMP AP second interval (mm)	6.0	2.2	5.1	1.4	5.7	1.4	4.1	1.1
CoP AMP AP third interval (mm)	5.5	1.4	4.3	1.0	5.1	1.2	3.7	1.3
CoP AMP ML first interval (mm)	8.2	2.3	6.6	1.8	8.7	2.1	6.2	1.5
CoP AMP ML second interval (mm)	8.2	2.9	6.9	1.8	8.3	3.7	5.3	2.0
CoP AMP ML third interval (mm)	7.5	2.2	5.7	1.7	7.6	3.2	5.1	1.9

CoP, centre of pressure; VEL, velocity; AMP, amplitude; AP, anterior-posterior; and ML, medial-lateral.

In contrast to DIF_31 for CoP AP amplitude, the DIF_21 for CoP ML amplitude decreased after fatigue for females (*p*=0.024; *d*=0.81), but not for males (*p*=0.533). This difference in females was a result in slightly increased CoP ML amplitude in first interval after fatigue (before fatigue: 6.6±1.8mm; after fatigue: 6.9±1.8mm) and concomitant drop during the second interval (before fatigue: 6.2±1.5mm; after fatigue: 5.3±2.0mm). Although no other statistically significant gender-specific effects were found, all data tended to indicate that after fatigue, males exhibit increased postural sway in the early phases of the trial and relatively unchanged postural sway in later phases of the trial. On the other hand, females decreased the postural sway in later phases of the trial, while their early postural sway was relatively unaffected by fatigue.

After fatigue, the correlation between whole-trial CoP ML velocity and its DIF_21 index (*r*=0.59; *p*=0.002) was still present and was similar in males only (*r*=0.55; *p*=0.043) and females only (*r*=0.57; *p*=0.034). There was also a correlation between whole-trial CoP ML amplitude and its DIF_21 index (*r*=0.65; *p*=0.001), which was also present both in males (*r*=0.76; *p*=0.002) and females (*r*=0.54; *p*=0.048).

## Discussion

The purpose of this study was to explore the effects of whole-body fatigue, induced by repeated CoD task on postural sway in single-leg stance and its transient characteristics. Our hypothesis was rejected, as the fatiguing protocol did not result in an increase of postural sway. On the contrary, postural sway was even reduced in females (the difference for CoP velocity, but not CoP amplitude being statistically significant), but did not change in males. Some of the indexes of transient postural sway behavior were influenced by fatigue. A closer examination of the data reveals that the changes are in accordance with our hypothesis, as the postural sway was more variable across trial after fatigue (in turn, the indexes of transient behavior were lower), with males being significantly affected in particular during the early phases of the trial. Although we expected larger deteriorations in postural sway, this study nevertheless generated two important findings. Firstly, the response to fatigue was time interval specific, which warrants further investigation of the transient behavior in relation to fatigue. Secondly, there were considerable differences between male and female participants in responses to fatigue, which points out that gender-specific effect of fatigue on postural sway.

Contrary to previous findings, some (positive) correlations were present between whole-trial-estimates and corresponding indexes of transient behavior. This implies that individuals with higher postural sway showed less stabilization (i.e., their sway was reduced less or was even increased throughout the trial). However, given that the associations were moderate, and the fact that four previous studies on larger sample sizes found no associations between whole-trial-estimates and corresponding indexes of transient behavior (Reed et al., 2020; Kozinc and Šarabon, 2021a,b; Kozinc et al., 2021), it is still reasonable to conclude that calculating and analyzing indexes of transient behavior can contribute new information to the traditional whole-trial analysis. Future studies investigating this topic should include correlational analysis to resolve this issue further.

The absence of the main effect of fatigue was a surprising finding, given that studies have reported increases in postural sway following aerobic and anaerobic (Fox et al., 2008; Güler et al., 2020), local and global (Zech et al., 2012), and sport-specific fatiguing (Greig and Walker-Johnson, 2007; Bedo et al., 2020). The effects of fatigue on postural sway could be related to reductions in muscle force producing capacity (Thorlund et al., 2008; Rampinini et al., 2011), which is observed after both central (Gandevia, 2001) and peripheral fatigue (Fitts, 1994). Other possible underlying causes of increases postural sway include changes in proprioception (Proske, 2019), and specifically for global fatigue, the perturbations introduced due to intensified breathing (Janssens et al., 2010). The most reasonable explanation for the lack of effect of fatigue on postural sway in the present study is that the fatiguing protocol was not sufficiently exhaustive to elicit the expected changes in males. The reduced postural sway in females after fatigue is the most difficult to interpret. The reductions of postural sway did not always imply better balance (Cho et al., 2014). It could be that females changed the postural control strategy. Anyway, while the type of fatiguing protocol is an important limitation of this study, at the same time, our results demonstrate that the indexes of transient behavior could perhaps detect smaller fatigue-induced changes that are masked by whole-trial estimates. Looking only at the whole-trial estimates, one could conclude that our protocol did not affect (males) or reduced (females) the postural sway, while the examination of interval-specific values showed increases sway (males) in the early phase of the trial. It is difficult to speculate what effect would be seen if a more exhausting protocol was used or if the participants were professional athletes. Likely, the effects of fatigue would be even smaller, considering that athletes’ balance is less influenced by fatigue compared to general population (Baghbani et al., 2016).

We observed considerable differences regarding the responses in postural sway to fatigue between males and females. Previous studies have suggested that there are no differences in postural sway between the genders in the adult population (Hageman et al., 1995; Cruz-Gómez et al., 2011; Olchowik et al., 2015), or that females exhibit lower postural sway (Greve et al., 2013; Błaszczyk et al., 2014; Sell et al., 2018). Smaller postural sway in females has been often attributed to lower body height (Era et al., 1996; Bryant et al., 2005). However, smaller postural sway in females has been found compared to males after adjusting for height (Frandin et al., 1995), and studies have reported no association between anthropometric variables and postural sway (Ekdahl et al., 1989; Ageberg et al., 2001). While the gender differences in postural sway in the rested state could be at least partially influenced by body height, the causes for different responses to fatigue are likely elsewhere. Masui et al. (2005) reported that the postural sway in older adult males was more influenced by the removal of visual information, compared to older adult females. However, since our fatiguing protocol presumably affected proprioception (leaving vison unperturbed), it would be expected that males would be less affected by fatigue if they placed more importance to visual information. One possible explanation for our results is that females are in general better in sensory integration and sensory reweighing, although the available data on adult populations suggest no gender differences (Faraldo-García et al., 2012). Studies in children suggest that females are better at integrating their sensory inputs, while males treat each sensory input more independently (Smith et al., 2012). It could be that when the balance is challenged (e.g., in fatigued conditions, and/or after the transition to a more challenging posture, such as single-leg stance), the females are better and quicker in adapting their sensory integration in order to stabilize the body. Bannon et al. (2018) observed statistically significant increases in postural sway in males, but not in females, after fatiguing load-lifting task. They concluded that females are able to better adapt their balance strategies to fatiguing conditions than males. Indeed, Adlerton et al. (2003) found increased CoP amplitude and trunk accelerations after local ankle fatiguing in females, whereas CoP velocity was decreased, reflecting a change in the postural control strategy.

Some limitations of the study should be acknowledged. The fatigue protocol was somewhat atypical in comparison to previous studies, which warrant further investigation with different (e.g., local and global, aerobic, and anaerobic) fatiguing protocols. The participants in our study were physically active young adults, which means that our results cannot be generalized to other populations such as elite athletes or older adults. In addition, it has to be stressed that our results cannot be extrapolated to the dynamic tasks. Namely, some of the previous studies have observed an effect of fatigue on static balance (i.e., increased postural sway in quiet stance; Zech et al., 2012) without concomitant changes in dynamic balance. Moreover, the contribution of individual sensory systems, as well the sensory integration, is probably dependent on the dynamics and difficulty of the task (Bent et al., 2002, 2005). Finally, the reliability of the interval-specific CoP data and indexes of transient behavior is questionable. It is known that trial duration increases the reliability of CoP metrics (Carpenter et al., 2001). Since only one trial was conducted for this study in each condition, poor reliability could have affected the results. While we cannot calculate the reliability for this specific sample, we found relatively high coefficients of variation (among three repetitions of the 30-s trial) on a sample of 73 male soccer players (unpublished data). Specifically, the coefficients of variation ranged from 9 to 14% for whole trial CoP velocity and amplitude variables, and from 12 to 27% for interval-specific data. Indeed, postural sway assessed through CoP movements is highly variable, and coefficient of variation >10% is often observed (Hébert-Losier and Murray, 2020). We used one repetition in our protocol because the fatigue could subside quickly after the completion of the fatiguing protocol (Aboodarda et al., 2019; Güler et al., 2020). Nevertheless, more repetitions should be used prior to the fatiguing protocol to increase the rigor of the results. Before the research on the transient behavior of postural sway is advanced, a methodological study is urgently needed to determine the influence of variable choice, postural task, and trial duration (as well as the duration of individual intervals, used to assess transient behavior) on within- and between-session reliability.

## Conclusion

The purpose of this study was to explore the effects of whole-body fatigue on postural sway in single-leg stance and its transient characteristics. Contrary to previous studies, postural sway did not increase with fatigue; rather it was even reduced in females. Some of the indexes of transient postural sway behavior were influenced by fatigue. Postural sway tended to be more variable across the trial after fatigue. This that the indexes of transient behavior could perhaps detect smaller fatigue-induced changes that are masked by whole-trial estimates. Males were particularly affected during the early phases of the trial. Thus, we can conclude that the response to fatigue in terms of postural sway is time interval specific, and that there are certain gender differences in responses to fatigue, which could be related to a better ability to adapt balance strategies in females. Transient characteristics of postural sway represent a potentially useful approach to analyze balance and postural control. Further studies will be needed to assess the utility of this approach for assessing sports performance and injury risk.

## Data Availability Statement

The raw data supporting the conclusions of this article will be made available by the authors, without undue reservation.

## Ethics Statement

The studies involving human participants were reviewed and approved by Republic of Slovenia National Medical Ethics Committee. The patients/participants provided their written informed consent to participate in this study.

## Author Contributions

ŽK, NT, and NŠ conceptualized the idea. NT and DS carried out the measurements. NŠ and DS were overviewing the measurement procedures and administration. NT and ŽK analyzed the collected data. ŽK wrote the manuscript. NŠ, NT, and DS finalized the manuscript. All authors contributed to the article and approved the submitted version.

## Conflict of Interest

NŠ was employed by company S2P, Science to Practice, Ltd.

The remaining authors declare that the research was conducted in the absence of any commercial or financial relationships that could be construed as a potential conflict of interest.

## Publisher’s Note

All claims expressed in this article are solely those of the authors and do not necessarily represent those of their affiliated organizations, or those of the publisher, the editors and the reviewers. Any product that may be evaluated in this article, or claim that may be made by its manufacturer, is not guaranteed or endorsed by the publisher.
